# Nutritional status deteriorates as the severity of diabetic foot ulcers increases and independently associates with prognosis

**DOI:** 10.3892/etm.2012.780

**Published:** 2012-10-30

**Authors:** SHAN-SHAN ZHANG, ZHENG-YI TANG, PING FANG, HONG-JIE QIAN, LEI XU, GUANG NING

**Affiliations:** Department of Endocrine and Metabolic Diseases, Rui-Jin Hospital, Shanghai Clinical Center for Endocrine and Metabolic Diseases, Shanghai Institute of Endocrine and Metabolic Disease, Shanghai Jiao-Tong University School of Medicine, Shanghai 200025, P.R. China

**Keywords:** nutritional status, diabetic foot ulcers, complication, infection

## Abstract

The prognosis for diabetic foot ulcers (DFUs) remains poor. Nutritional status has not been identified as one of the factors affecting the outcome of DFUs. Therefore, indicators correlated with nutritional status and outcome were analyzed to investigate their relationship. A total of 192 hospitalized patients with Wagner grade 1–5 ulcers and 60 patients with Wagner grade 0 ulcers (all had type 2 diabetes) were assessed by the following: subjective global assessment (SGA), anthropometric measurements, biochemical indicators and physical examinations to evaluate nutritional status, severity of infection and complications. Patient outcome was recorded as healing of the ulcer and the patients were followed up for 6 months or until the wound was healed. The percentage of malnutrition was 62.0% in the DFU patients. The SGA was closely correlated with infection (r=0.64), outcome (r=0.37) and BMI (r=−0.36), all P<0.001. The risk of poor outcome increased with malnutrition [odds ratio (OR), 10.6, P<0.001]. The nutritional status of the DFU patients was independently correlated with the severity of infection and outcome (both P<0.001) and Wagner grades and nutritional status (SGA) were independent risk factors for patient outcome (both P<0.001). Nutritional status deteriorated as the severity of the DFU increased, and malnutrition was a predictor of poor prognosis.

## Introduction

The number of patients with diabetes mellitus (DM) has been increasing rapidly worldwide; the prevalence of DM has increased from 2.5% in 1994 to 9.7% in 2008 in China (1,2), and the incidence of diabetic foot ulcers (DFUs) has increased concurrently (3). The prognosis for DFUs remains poor, although our understanding and treatment of this late-stage complication of DM have improved (4). As a common and serious complication of diabetes, DFUs are associated with significant mortality (5).

Patients with DM are generally considered to have excessive food intake and medical nutrition therapy is required to aid individuals with diabetes to achieve blood glucose targets (6). Whereas previous studies have shown that the nutritional status of patients in surgery, who are critically ill, have cancer or end-stage renal disease, is markedly correlated with the development of complications, hospital stay, life expectancy and total outcomes (7–10); when combined with diabetes, all the above became worse. In clinical practice, fewer DFU patients achieved blood glucose targets and a greater number suffer from vascular complications and infection, or reveal a poor nutritional status compared with non-DFU patients. These cause greater difficulties in treatment; calorie intake should be restricted to achieve targets for blood glucose and related metabolic markers while protein intake should be confined to reduce proteinuria and improve the prognosis for diabetic nephropathy (DN). However, the additional energy expenditure due to infection requires increased energy intake, and following surgery, patients require sufficient nutrients to recover (11).

Weight loss during the course of diabetes reflects changes in body composition and function, although in the context of diabetes, this may also be a sensitive signal of nutritional depletion. Sohn *et al*(12) reported a significant J-shaped association between BMI and DFU and Yekta *et al*(13) reported that a BMI<25 kg/m^2^ was significantly associated with amputation. In the present study, the mean BMI was 22.1 kg/m^2^ in the Wagner grade 1–5 patients and decreased as the severity progressed; this value is lower than that of patients at the time of diagnosis of DM in China (2). Together with decreasing BMI, vascular complications, including neuropathy, nephropathy and peripheral vascular disease (PVD) and certain nutritional indicators (hemoglobin, serum albumin, total cholesterol) deteriorated gradually. Moreover, these indicators were more serious in patients with Wagner grade 4 and 5 ulcers. Our study hereby suggests that the treatment of DFU should focus on foot management together with improvements of general status, including the amelioration of nutritional status.

## Subjects and methods

### Subjects

All subjects (192 DFU cases with Wagner grade 1–5 ulcers and 60 cases with Wagner grade 0 ulcers, all with type 2 diabetes) were hospitalized between January and December 2009. The study protocol was approved by the Institutional Review Board of Rui-Jin Hospital and the patients had provided informed consent. To be eligible for inclusion, patients had to undergo a foot examination and be definitively diagnosed with DFU. The severity of the foot disease was graded according to Wagner’s classification (14), which assesses ulcer depth and the presence of osteomyelitis or gangrene using the following grades: grade 0 (pre-or postulcerative lesion), grade 1 (partial/full thickness ulcer), grade 2 (probing to tendon or capsule), grade 3 (deep with osteitis), grade 4 (partial foot gangrene) and grade 5 (whole foot gangrene). All patients were interviewed individually to obtain information concerning their medical histories. Anthropometrics, evaluation of nutrition status, assessment of diabetic complications and comorbidities and foot-specific information at presentation were recorded, and patients were followed up until the would was healed or for 6 months.

### Measurements and evaluation of nutrition status and clinical indicators

For anthropometrics, height and weight were measured with light clothes and without shoes by the same physician. BMI was calculated as the body weight in kilograms divided by the height squared in meters. Blood pressure was measured at the right arm with an automated electronic device (OMRON Model 1 plus; Omron Company, Kyoto, Japan) three times consecutively at 1 min intervals following at least 15 min rest in the seated position or in bed; the three readings were averaged for analysis. A subjective global assessment (SGA) was performed by a trained independent physician within 72 h of admission, based on medical history and physical examination, including changes in weight, dietary intake, functional capacity, gastrointestinal symptoms, metabolic stress, loss of subcutaneous fat, muscle wasting and ankle/sacral edema. This information was used to classify patients into one of three categories of nutritional status: A, well nourished; B, moderately malnourished; or C, severely malnourished (15). Nutrition status was evaluated from the SGA, BMI and hemoglobin, total protein, serum albumin and total cholesterol levels.

The serum concentrations of triacylglycerol, total cholesterol, total protein and albumin were detected using an autoanalyser (Hitachi 7080 Automatic Analyzer; Hitachi Science Systems, Ltd., Ibaraki, Japan). Hemoglobin was measured by chromatometry using automatic equipment (ABX Pentra 80; Horiba, Montpellier, France). The 24 h-urine protein was measured by pyrogallol red molybdenum chromatometry (Dxl 800; Beckman Coulter, Miami, FL, USA) and 24 h-urine microalbumin was tested using immunoturbidimetry (Dade Behring BN II; Siemens, Munich, Germany). HbA1c was analyzed by high pressure liquid chromatography with the BioRad Variant Hemoglobin HbA1c assay (Hercules, CA, USA).

### Assessment of diabetic complications and comorbidities

Patients were screened for microalbuminuria by measuring albumin from a 24-h urine collection. A urinary albumin level of >30 mg per 24 h was diagnostic using a timed accumulated sample. According to American Diabetes Association guidelines, DN is diagnosed if two of three tests for microalbuminuria are positive in a three- to six-month period (16), excluding transient albuminuria caused by exercise, urinary tract infections, hyperglycemia, febrile illness, severe hypertension or heart failure. Patients with overt nephropathy were detected easily by routine urinalysis, a urinary albumin level of >300 mg per 24 h or medical record.

Patients were identified and categorized for diabetic peripheral neuropathy using a clinical examination and conventional nerve conduction studies, or had been definitively diagnosed previously. Based on the American Academy of Neurology criteria, the classification of neuropathy is based on the presence of at least one neuropathic symptom or sign together with electrophysiological polyneuropathy as defined by an abnormality of at least two parameters in at least two nerves (17). Other causes of the neuropathy should be excluded for the diagnosis to be made.

PVD, commonly referred to as peripheral arterial disease or peripheral artery occlusive disease, is the obstruction of large arteries not within the coronary or aortic arch vasculature or the brain. In the current study, PVD was examined only in the lower extremities. Patients with calcified arteries from DM occasionally have relatively non-compressible arteries leading to falsely elevated ankle/brachial index (ABI) values in the normal range. Thus, in our study, PVD was considered to be present if the patients had acute or critical limb ischemia, or intermittent claudication; it was documented in the medical record or there was a history of limb revascularization (18); posterior tibialis and dorsalis pedis pulses to palpation in the same limb were absent or diminished (19); or stenosis or obliteration of the lower extremity arteries was identified following examination by doppler ultrasonography, computed tomographic angiography, magnetic resonance angiography or contrast arteriography.

Ulcers or gangrene were determined to be infected if a purulent discharge and two other local signs (warmth, erythema, lymphangitis, lymphadenopathy, edema or pain) were present (20). The severity of infection was evaluated according to the clinical classification of diabetic foot infection instituted by the Infectious Diseases Society of America (21). The classification was described briefly as: uninfected, wound lacking purulence or any manifestations of inflammation; mild, presence of ≥2 manifestations of inflammation (purulence, or erythema, pain, tenderness, warmth or induration), but any cellulitis/erythema extends ≤2 cm around the ulcer, and infection is limited to the skin or superficial subcutaneous tissues, no other local complications or systemic illness; moderate, infection (as above) in a patient who is systemically well and metabolically stable but which has at least one of the following characteristics: cellulitis extending >2 cm, lymphangitic streaking, spread beneath the superficial fascia, deep-tissue abscess, gangrene and involvement of muscle, tendon, joint or bone; severe, infection in a patient with systemic toxicity or metabolic instability (for example, fever, chills, tachycardia, hypotension, confusion, vomiting, leukocytosis, acidosis, severe hyperglycemia or azotemia).

### Treatment of patients with foot ulcers and outcome assessment

On the basis of the results assessed by general and foot status, treatments were given individually. In general, for foot ulcer care, patients were treated with insulin to control blood glucose, hemorheologic agents and trophic nerve agents to improve foot blood supply *pro re nata*, antibiotics when infected, debridement or part amputation when abscess or gangrene was present, or blood or albumin infusion if severe anemia or hypoproteinemia existed, without interventional treatment.

The patients’ outcomes over the 6 months were defined as healing (ulcer healed), deferment (ulcer did not heal), recurrence (ulcer recurred), above-ankle amputation or mortality.

### Statistical analysis

Data are expressed as the mean and standard error (continuous variables) or as a number and percentage (categorical variables). Measurements with a skewed distribution were normalized by logarithmic transformation. Comparisons of means and proportions were performed with an ANOVA or χ^2^ test, as appropriate. The homogeneity of groups was determined when the means had significant differences. Fisher’s least significant difference (LSD) post hoc test was applied for multiple comparisons where appropriate. Spearman’s rank correlation analysis was used to examine the relationship between SGA and potential affecting factors. To assess the potential association between SGA and the number of ulcers not healed by the end of the study period, a χ^2^ analysis with odds ratio (OR) was performed. Multiple stepwise regression analysis was conducted to examine the main factors affecting nutrition status and outcome. SPSS 13.0 for Windows (SPSS Inc., Chicago, IL, USA) was used for all analyses. P<0.05 was considered to indicate a statistically significant result.

## Results

### Clinical characteristics of DFU patients with various Wagner grades

Table I shows the baseline demographic details for the group of patients at first presentation. For the 192 patients in the Wagner grade 1–5 groups, the mean age and duration of DM were 68.6±11.3 and 12.3±8.1 years, respectively. Most of these patients had poor blood glucose control (mean HbA1c was 8.8%). Indicators of nutritional status (BMI, albumin, total protein, hemoglobin and total cholesterol) were lower than those in patients with Wagner grade 0 ulcers. Following ANOVA adjustment for age, gender, duration of DM and duration of DFU, the patients with Wagner grade 4 and 5 ulcers had significantly lower cholesterol levels, BMI (also adjusted for SBP and DBP), hemoglobin levels (also adjusted for HbA1c, total protein, creatinine and 24 h-urine protein) and albumin levels (also adjusted for total protein, creatinine, 24 h-urine protein and 24 h-urine microalbuminuria) than the patients with Wagner grade 0 and 1 ulcers (all P<0.05). The percentages of DFU patients who had diabetic peripheral neuropathy, diabetic nephropathy, peripheral vascular disease and infection at presentation were 84.4, 45.3, 74.5 and 83.9% (161/192), respectively. As the Wagner grade increased from 0 to 5, the percentages of these complications and comorbidities increased.

### Nutritional status of patients with DFU

**Figure 1:**
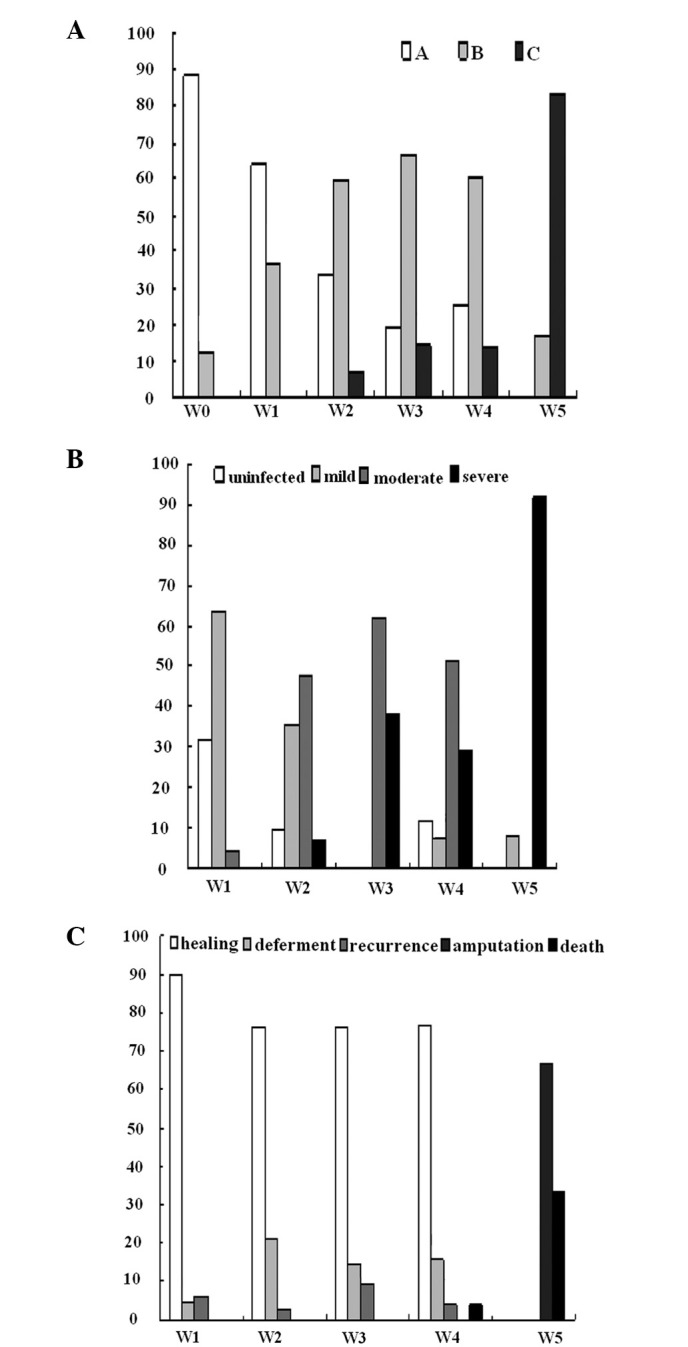
As the Wagner (W) grades increased, nutritional status, severity of infection and outcome deteriorated. (A) Nutritional status in patients with ulcers of Wagner grades 0–5. The prevalence of malnutrition was 62.0% in patients with foot ulcers. (B) Severity of infection in patients with ulcers of Wagner grades 1–5. Infection was identified in 83.9% of the DFU patients. (C) Outcome of patients with ulcers of Wagner grades 1–5. Only a small proportion of patients with ulcers of Wagner grades 1–4 did not heal well, while the outcome of patients with grade 5 ulcers was poor. (A–C) P<0.001 for the χ^2^ analysis across all groups. DFU, diabetic foot ulcer.

Only 11.7% of patients with Wagner grade 0 ulcers were malnourished (SGA-B or SGA-C) compared with 62.0% of patients with Wagner grade 1–5 ulcers at presentation. As the Wagner grade increased, the percentage of malnutrition also increased (Fig. 1A).

Even at same levels of age, duration of DM and HbA1c, the indicators associated with nutrition differed among the patients in each of the SGA groups (Table II). Along with deteriorating nutritional status, patients presented a longer duration of DFU, higher serum creatinine levels and more protein leakage.

### Severity of infection in patients with DFU

The percentage of DFU patients who had clinically infected ulcers at presentation was 83.9% (68.2–100% from Wagner grades 1 to 5) and the incidence of moderate and severe infection increased for Wagner grades >3 (Fig. 1B). Due to mummification necrosis, some patients with Wagner grade 4 and 5 ulcers were identified to be uninfected or mildly infected.

### Outcomes of DFU patients and interactions of nutrition, infection and outcome

At the end of the study period, the majority of ulcers of Wagner grade 1–4 were healed (146/180, 81.1%) and no patient required amputation above the ankle. The percentages of ulcer deferment or recurrence were also relatively low among these grades (Fig. 1C). Two of the 51 patients with Wagner grade 4 ulcers unexpectedly succumbed following 10 and 28 days hospitalization. For patients with Wagner grade 5 ulcers, the outcome was either above-ankle amputation or mortality.

Retrospective analysis identified that the DFU patients with various outcomes had significant differences in BMI, total protein, serum albumin, hemoglobin and HbA1c at first presentation. The poorer the outcome, the worse these factors, despite age, duration of DM and duration of DFU among these groups being at similar levels (Table III).

**Figure 2:**
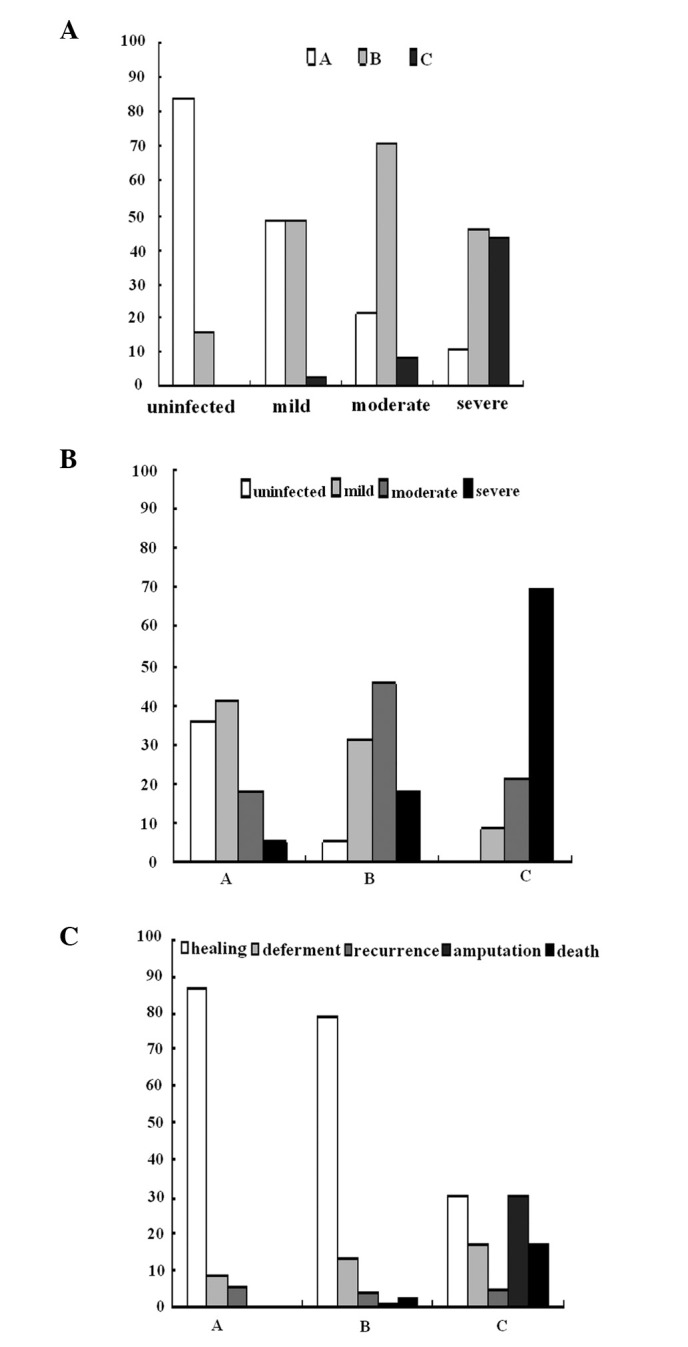
Interactions of nutritional status, severity of infection and outcome. (A) Severity of infection correlated with the nutritional status. None of patients with uninfected feet were severely malnourished; only a small proportion of the mildly or moderately infected patients were severely malnourished, however, 43.2% of the patients with severe infection were severely malnourished. (B) Nutritional status associated with the severity of infection. Severe infection was observed in a small proportion (5.5%) of the well-nourished (A) patients, was 3-fold higher (17.7%) in the moderately malnourished (B) patients and 69.6% in the severely malnourished (C) group. (C) Outcome varied with nutritional status. The majority of foot ulcers in well nourished patients healed. In moderately malnourished patients, the proportion of deferment or recurrence was not large. Severely malnourished patients had high rates of mutilation and mortality. P<0.001 for the χ^2^ analysis across all groups.

None of the DFU patients with uninfected feet were severely malnourished, the majority of the patients with mildly or moderately infected feet were moderately malnourished, while 43.2% of patients with severe infection were severely malnourished (Fig. 2A). However, few well-nourished patients developed moderate or severe infection, whereas 69.6% of severely malnourished patients were severely infected (Fig. 2B). The majority of the foot ulcers in the well nourished patients healed (86.3%), but those in malnourished patients tended to deferment or recurrence. Patients with SGA-C status had poor outcome (69.6%) with high rates of mutilation (7/23, 30.4%) and mortality (4/23, 17.4%; Fig. 2C).

Malnourished patients (SGA-B and -C) were 11-fold more likely to have a poor outcome (not healed in six months) than SGA-A patients (69.6 vs. 17.8%; P<0.001; OR, 0.6; 95% CI, 4.1–28.0).

### Correlation analysis and multiple stepwise regression analysis of SGA, outcome and correlated factors

The SGA result significantly correlated with duration of DFU, infection status, Wagner grades, BMI, urine protein leakage and outcome, all P<0.05. Multiple stepwise regression analysis identified that the severity of infection and outcome were independently associated with the patients’ nutritional status, and the standardized coefficient or β values were 0.47 and 0.28 respectively, both P<0.001. Analysis also revealed that the independent risk factors of outcome were severity of DFU (Wagner grades, β=0.33) and nutritional status (SGA, β=0.28), both P<0.001.

## Discussion

Few studies have identified foot ulcer classification systems as predictors of clinical outcome. The current study not only assessed these factors, but also was the first to identify nutritional status as a predictor for the clinical outcome of DFU patients. As the Wagner grade of the ulcer increased: the BMI and serum albumin, hemoglobin and total cholesterol levels decreased; the urine protein leakage, severity of infection and percentages of SGA grades B and C increased; and nutritional status deteriorated. The patients’ outcome was independently affected by the severity of the DFU and nutritional status. Malnutrition was identified in 62.0% of the studied patients and malnutrition at presentation was predictive of poor outcome.

Poor nutritional status is significant in the prognosis of most chronic, critical or infectious diseases, or following surgery (7–10). As in uremic diabetic patients, nutritional indicators, including age, BMI and low serum albumin concentrations, were independent factors associated with mortality, initiated time to dialysis and other complications (10,22). For DFU patients, the factors affecting outcome include Wagner grades, BMI, serum albumin and, more importantly, infection and nutritional status; the affect of nutritional status is similar in other diseases.

A number of factors are involved in and lead to malnutrition in patients with DFUs. A higher resting energy expenditure (REE) may contribute to the deterioration in nutritional status of the diabetic patients with foot ulcers, since type 2 DM mirrors chronic disease states associated with elevated protein turnover and rapid loss of body protein (23). The kinetics of whole-body protein metabolism were elevated and net balance was diminished. Elevated flux has been identified to be associated with increased REE, insulin resistance or lack of insulin secretion; these alterations were worsened with the magnitude of hyperglycemia (24). DFU patients may expend more energy and lose more protein than non-DFU patients due to elevated flux and poorly controlled blood glucose.

Hyperglycemia and a negative nitrogen balance cause diabetic patients to tend to malnutrition and infection. With skin damage and poor blood supply, DFU patients have a very high rate of infection; it was 83.9% in the current study, and a high proportion of the ulcers were moderately or severely infected. Infection and malnutrition have always been intricately linked (25). The interaction of the two leads to a synergistic vicious cycle of increased susceptibility to infection and adverse nutritional status (26).

According to our data and previous studies (4,19,27,28), DFU patients have a long duration of DM, accompanied by a high morbidity rate of micro- and macro-vascular complications. Diabetes is often associated with nephropathy, which is a disturbance involving protein leakage and a decreased glomerular filtration rate, and peripheral neuropathy, including autonomic neuropathy which gives rise to pain, numbness, gastroparesis, diarrhea and movement intolerance. These, together with vascular complications, result in sleeplessness, poor appetite, increased energy expenditure, loss of protein, edema and deteriorating nutritional status.

SGA is used primarily by clinicians to assess the nutritional status of hospitalized patients. Compared with the Nutritional Risk Index (serum albumin and recent weight loss), BMI and serum albumin, SGA acted as a good predictor for malnutrition and complications (10,29). In the current study, most DFU patients with severe malnutrition had a poor prognosis: 17.4% deferment, 30.4% above-ankle amputation, 17.4% mortality and only 30.4% healing, whereas for the moderately malnourished patients, the ulcer healing rate sharply increased to 79.2%, and further increased to 86.3% in patients with a well-nourished status. Malnutrition has a marked association with increased risk of poor outcome and predicts poor ulcer healing.

If DFU patients with severe malnutrition receive sufficient nourishment, their prognosis may be improved. This is clearly positive, but in chronic diseases, it may not be possible for patients with severe malnutrition to be provided with large amounts of calories in a short time, or quickly infused with blood products to elevate hemoglobin or serum albumin to normal levels (30,31), as the treatment may result in further complications, including heart failure and impairment of renal function. The outcome data in the current study were acquired following proper nutritional supplementation, and our results suggest that even with the appropriate treatments for general condition, including nutrition amelioration, infection control and foot care, the prognosis of severely malnourished patients remains poor.

Certain flaws of the current study should be addressed. The small sample size in the SGA-C, Wagner grade 5 and certain outcome groups resulted in an unbalanced distribution of some clinical indicators in different groups, which may limit the power of data analysis. Increasing the number of DFU cases may improve this defect, but data collected over a longer time or from other centers may affect the consistency of the results due to inequalities in the tests and treatments. The clinical characteristics of the subjects in the current study differed from those in a number of previous studies (32,33). Our patients were older, with longer durations of DM and DFU, more abnormalities of biochemical indicators and higher percentages of complications and comorbidities. These differences may be due to diversities of the diagnostic tests and population selection.

In conclusion, the higher the Wagner grade, the poorer the nutritional status and outcome. Malnutrition was common in DFU patients, and the prognosis of the severely malnourished patients was poor, despite compensation with appropriate treatments. Assessment of the nutritional status of DFU patients should be emphasized since it is a key anticipator of outcome.

## Figures and Tables

**Table I:** Clinical characteristics of patients grouped by Wagner’s classification.

Variables	W0	W1	W2	W3	W4	W5	(W1–5)	Total	Group comparison	
									Statistics	P-value
Total (N)	60	66	42	21	51	12	192	252		
Gender, male/female	37/23	39/27	28/14	15/6	30/21	6/6	118/74	155/97		
Age, years	67.5±12.3	69.2±11.0	68.8±10.2	64.4±11.8	68.8±11.5	71.7±14.3	68.6±11.3	68.4±11.5	0.858	0.510
BMI, kg/m^2^	23.7±3.3	22.9±3.6	22.3±3.2	21.5±2.2^a^	21.9±2.9^a^	18.6±1.9^a–e^	22.1±3.2	22.5±3.3	11.329	0.000
Duration of DM, years	11.3±9.5	12.2±8.8	14.2±7.9	12.7±7.9	11.0±7.1	9.8±7.9	12.3±8.1	12.0±8.4	1.019	0.407
Duration of DFU, days	/	53±105	129±250	67±86	72±73	70±50	77±143	/	1.977	0.100
HbA1c, %	8.5±2.1	8.2±2.0	8.7±2.0	8.8±1.9	9.0±2.4	11.5±2.4^a–e^	8.8±2.2	8.7±2.2	4.597	0.001
Triglyceride, mmol/l	1.65±1.31	1.65±1.44	1.41±1.47	1.07±0.50	1.10±0.34^a^	0.97±0.41^a^	1.35±1.15	1.43±1.19	4.031	0.003
Total cholesterol, mmol/l	4.57±1.10	4.35±1.19	4.25±1.13	3.89±1.04^a^	3.72±1.24^ab^	3.53±1.42^a,b^	4.07±1.21	4.19±1.20	3.979	0.002
Hemoglobin, g/l	123.0±14.5	117.3±17.8	111.3±20.4^a^	106.1±15.0^a,b^	103.2±16.5^a–c^	91.8±23.0^a–e^	109.4±19.4	112.7±19.2	12.295	0.000
Total protein, g/l	65.9±6.8	66.1±5.9	64.2±7.1	65.2±8.3	65.5±6.1	57.2±8.1^a–e^	64.9±6.9	65.1±6.9	3.941	0.002
Albumin, g/l	37.9±3.7	36.9±3.9	34.3±4.7^a,b^	32.0±5.5^a–c^	32.8±4.2^a,b^	25.2±5.4^a–e^	34.0±5.3	34.9±5.2	25.935	0.000
Creatinine, μmol/l	73.0±34.0	84.1±43.5	76.8±30.9	84.2±57.7	79.9±38.1	92.8±70.1	82.0±43.4	79.8±41.5	0.686	0.636
24 h-urine protein, mg/24 h	273.3±401.2	540.4±1025.7^a^	897.5±1271.7^a^	911.5±1935.8^a^	772.4±1076.7^a^	1043.8±850.9^a^	752.9±1212.5	622.3±1075.6	5.493^f^	0.000^f^
Diabetic nephropathy, n/N (%)	21/60 (35.0)	20/66 (30.3)	20/42 (47.6)	10/21 (47.6)	27/51 (52.9)	10/12 (83.3)	87/192 (45.3)	108/252 (42.9)	16.489	0.006
Diabetic peripheral neuropathy, n/N (%)	27/60 (45.0)	48/66 (72.7)	38/42 (90.5)	20/21 (95.2)	45/51 (88.2)	11/12 (91.7)	162/192 (84.4)	189/252 (75.0)	45.477	0.000
Peripheral vascular disease, n/N (%)	25/60 (41.7)	37/66 (56.1)	36/42 (85.7)	17/21 (81.0)	42/51 (82.4)	11/12 (91.7)	143/192 (74.5)	168/252 (66.7)	38.024	0.000

W, Wagner classification; BMI, body mass index; DM, diabetes mellitus; DFU, diabetic foot ulcer. Values are mean ± SD or number (proportion), P-values are for the ANOVA or χ^2^ analysis across the six groups.

a–e P<0.05 vs. the W0, W1, W2, W3 and W4 groups respectively.

f Values are for post logarithmic transformation.

**Table II:** Clinical characteristics of studied patients grouped by SGA.

Variables	SGA-A	SGA-B	SGA-C	Group comparison	
				Statistics	P-value
Total (N)	126	103	23		
Gender, male/female	78/48	64/39	13/10		
Age, years	67.2±11.6	69.5±10.5	69.7±14.7	1.246	0.289
BMI, kg/m^2^	23.5±3.2	21.8±3.1	19.5±2.3	19.725	0.000
Duration of DM, years	11.6±8.3	13.1±8.8	9.9±6.5	1.732	0.179
Duration of DFU, days^a^	55±87	73±101	138±292	3.312	0.039
HbA1c, %	8.7±2.1	8.5±2.2	9.6±2.9	1.227	0.302
Triglyceride, mmol/l	1.53±1.21	1.39±1.27	1.00±0.39	1.726	0.180
Total cholesterol, mmol/l	4.30±1.15	4.20±1.24	3.49±1.17	4.006	0.019
Hemoglobin, g/l	118.7±16.5	109.5±18.5	93.7±21.1	21.909	0.000
Total protein, g/l	66.1±7.0	65.1±5.8	59.5±8.5	9.591	0.000
Albumin, g/l	36.6±4.5	34.6±4.1	27.2±6.2	26.725	0.000
Creatinine, μmol/l	75.3±32.4	83.0±44.9	90.4±63.5	1.502	0.232
Log_10_UP-24h^b^	2.32±0.48	2.46±0.57	2.90±0.47	8.692	0.000
Log_10_UMA-24h^b^	1.74±0.60	1.87±0.76	2.20±0.58	3.038	0.050

Values are the mean ± SD or number (proportion). P-values are for the ANOVA across the three groups.

a Values are for 192 Wagner grade 1–5 patients.

b UP-24h, 24 h-urine protein; UMA-24h, 24 h-urine microalbumin. SGA, subjective global assessment; DM, diabetes mellitus; DFU, diabetic foot ulcer.

**Table III:** Clinical characteristics of DFU patients grouped by outcome.

Variables	Healing	Deferment	Recurrence	Amputation	Mortality	Group comparison	
						Statistics	P-value
Total	146	23	9	8	6		
Gender, male/female	92/54	13/10	7/2	5/3	1/5		
Age, years	68.1±11.5	72.7±8.4	64.3±8.6	67.1±12.3	74.2±15.6	1.540	0.192
BMI, kg/m^2^	22.6±3.2	20.9±2.6	21.6±3.1	18.3±1.8	20.5±3.3	4.992	0.001
Duration of DM, years	11.6±7.8	16.1±8.5	13.2±7.6	11.4±8.5	12.1±10.7	1.623	0.170
Duration of DFU, days	77±158	101±96	29±46	80±48	57±45	0.437	0.482
HbA1c, %	8.7±2.2	7.6±1.2	8.8±1.7	10.5±2.7	11.9±2.8	4.941	0.001
Triglyceride, mmol/l	1.44±1.27	1.05±0.45	1.24±0.55	0.99±0.47	0.98±0.14	0.878	0.479
Total cholesterol, mmol/l	4.18±1.24	3.67±0.89	4.03±0.94	3.56±1.58	3.30±0.55	1.529	0.196
Hemoglobin, g/l	112.2±18.3	101.5±17.5	109.0±22.7	84.3±24.4	104.0±9.9	5.682	0.000
Total protein, g/l	65.8±6.8	63.0±5.2	63.4±3.7	57.4±4.4	60.1±12.4	4.468	0.002
Albumin, g/l	35.0±4.9	32.8±2.9	31.7±3.5	23.9±4.6	29.3±6.2	13.276	0.000
Creatinine, μmol/l	80.0±41.2	82.1±34.7	76.8±29.4	88.3±72.1	127.8±76.6	0.573	0.686
Log_10_UP-24h	2.45±0.54	2.72±0.55	2.93±0.35	2.79±0.66	2.73±0.70	0.728	0.574
Log_10_UMA-24h	1.82±0.68	2.17±0.88	2.35±0.70	2.24±0.56	2.06±0.66	1.026	0.397

Data from 192 patients with Wagner grade 1–5 ulcers. Values are the mean ± SD or number (proportion), P-values are for the ANOVA across the five groups. DFU, diabetic foot ulcer; DM, diabetes mellitus; UP-24h, 24 h-urine protein; UMA-24h, 24 h-urine microalbumin.
